# Qualitative study to explore the health and well-being impacts on adults providing informal support to female domestic violence survivors

**DOI:** 10.1136/bmjopen-2016-014511

**Published:** 2017-03-24

**Authors:** Alison Gregory, Gene Feder, Ann Taket, Emma Williamson

**Affiliations:** 1 Centre for Academic Primary Care, University of Bristol, Bristol, UK; 2 School of Health and Social Development, Deakin University, Burwood, Victoria, Australia; 3 Centre for Gender and Violence Research, University of Bristol, Social Science Complex, Bristol, UK

**Keywords:** TRAUMA MANAGEMENT, MENTAL HEALTH, PRIMARY CARE, PSYCHIATRY, PUBLIC HEALTH

## Abstract

**Objectives:**

Domestic violence (DV) is hazardous to survivors' health, from injuries sustained and from resultant chronic physical and mental health problems. Support from friends and relatives is significant in the lives of DV survivors; research shows associations between positive support and the health, well-being and safety of survivors. Little is known about how people close to survivors are impacted. The aim of this study was exploratory, with the following research question: what are the health and well-being impacts on adults who provide informal support to female DV survivors?

**Design:**

A qualitative study using semistructured interviews conducted face to face, by telephone or using Skype. A thematic analysis of the narratives was carried out.

**Setting:**

Community-based, across the UK.

**Participants:**

People were eligible to take part if they had had a close relationship (either as friend, colleague or family member) with a woman who had experienced DV, and were aged 16 or over during the time they knew the survivor. Participants were recruited via posters in community venues, social media and radio advertisement. 23 participants were recruited and interviewed; the majority were women, most were white and ages ranged from mid-20s to 80.

**Results:**

Generated themes included: negative impacts on psychological and emotional well-being of informal supporters, and related physical health impacts. Some psychological impacts were over a limited period; others were chronic and had the potential to be severe and enduring. The impacts described suggested that those providing informal support to survivors may be experiencing secondary traumatic stress as they journey alongside the survivor.

**Conclusions:**

Friends and relatives of DV survivors experience substantial impact on their own health and well-being. There are no direct services to support this group. These findings have practical and policy implications, so that the needs of informal supporters are legitimised and met.

**Strengths and limitations of this study**

- This study provides an indepth exploration of the health and well-being impacts experienced by friends and family members supporting a woman who is experiencing domestic violence (DV).
- A key strength of this research is the novelty of perspective, because it accessed the experiences of friends and relatives directly, which is vital if we are to understand the wider context and implications of DV.
- The data came from face-to-face interviews, but the main researcher (AG) also kept a reflective diary and fieldnotes. AG also carried out a member-checking process during the interviews to increase rigour and validity of results.
- This study shows that friends, colleagues and relatives of survivors experience substantial impact on health and well-being and may, in some cases, be experiencing secondary traumatic stress.
- One of the limitations of this research was that the sample lacked breadth, particularly in terms of ethnicity. It will be important to try to address this in future research.

## Introduction

Domestic violence (DV) is a global issue to which no age group, culture or socioeconomic group is insusceptible.^1^ The United Nations Development Fund for Women estimates that, throughout the world, one in three women will experience violence in their lifetime, and in most cases, the abuser will be a family member.^2^

The Council of Europe, the WHO and the United Nations have all identified violence against women as a major public health issue.^2–4^ The most obvious health consequence is physical injury, with 70% of DV incidents resulting in injury.^5^ Less apparent are chronic health problems which result; research demonstrates links between DV and gynaecological problems,^6^ chronic pain,^7^ gastrointestinal disorders^8^ ^9^ and cardiovascular conditions.^10^ There is also substantial evidence for the harmful consequences on mental health, with depression, anxiety, post-traumatic stress disorder (PTSD), substance abuse and suicidal ideation commonly experienced by survivors.^11^ ^12^

Research suggests that the majority of female DV survivors choose to access support (practical and emotional) from adults around them.^13–16^ In a study by Parker and Lee,^14^ 89% of DV survivors disclosed the abuse they were experiencing to friends and relatives. While many survivors rely on informal support alongside professional and specialist services, there are a large number who rely initially, predominantly or exclusively on friends, relatives and colleagues.^13^ ^16^ ^17^ Research has demonstrated that positive social support buffers against effects of abuse on survivors' physical health, mental health and quality of life, and that it can be preventive against them experiencing further abuse.^18–21^

Exposure to violence can be traumatic in its own right.^22–24^ Indeed, the most recent edition of the Diagnostic and Statistical Manual of Mental Disorders (DSM-5)^25^ recognises the experiences of people who have witnessed traumatic events, and those who have learnt about events that have happened to close relatives and friends. Historically, this idea of secondary traumatic stress (STS)—sometimes referred to as indirect trauma, compassion fatigue or vicarious trauma—has only been applied to people working as professionals with traumatised patients or clients. More recently, however, researchers have begun to direct attention towards those providing support in an *informal* capacity, noting the overlap with impacts that professionals in caring roles experience.^26^

In summary, there is substantial evidence that women experiencing DV draw support from people in their social network and that, when this is positive, there are important benefits. However, because the *direct* study of people in DV survivors' social networks is rare, little is known about the possible diffusion of impacts, including the possibility of STS.^17^ ^27^ ^28^

## Methods

This qualitative study was conducted in the UK. The aim of the research was exploratory, with the following research question: what are the health and well-being impacts on adults who provide informal support to female DV survivors? Owing to the emotive nature of the topic, individual interviews were considered the most appropriate mode of data collection.

### Participants

Maximum variation sampling was used to recruit participants with a range of experiences, attitudes and beliefs. It is an approach which aims to capture and describe themes that cut across a great deal of participant variation, so that common patterns that *do* emerge are of value and interest. In order to access a diverse range of people, advertisement of the study included: posters in local healthcare and community settings, social media and web-advertisement, and promotion on local radio. Particular emphasis was placed on attempting to recruit participants with an ethnic background other than White British, in recognition of the general under-representation of individuals from minority ethnic backgrounds in health research.^29^ For this reason, the study was also advertised by agencies in Bristol working with black and minority ethnic groups.

Participants were eligible if they had had a close relationship with a female survivor of DV, and were aged 16 or over during the time they knew the survivor.

DV was defined according to the UK Home Office definition:

Any incident or pattern of incidents of controlling, coercive, threatening behaviour, violence or abuse between those aged 16 or over who are, or have been, intimate partners or family members regardless of gender or sexuality. The abuse can encompass, but is not limited to: psychological, physical, sexual, financial & emotional.^30^

Owing to the gender asymmetry around DV, particularly in terms of impact,^31–33^ and because much less is known about the ways men experiencing DV interact with their social networks,^34^ ^35^ it was decided to focus this work on the friends and relatives of *female* survivors, though the perpetrator could be of either gender.

Twenty-three participants were recruited and interviewed. A further 63 people expressed an interest in the study: 33 were ineligible (27 were survivors rather than informal supporters, 5 had been exposed to DV during childhood rather than adulthood, and 1 was not based in the UK), 28 made no further response after initial contact and 2 were recruited but failed to attend the arranged interview. The relationships that participants had to a survivor were: mother (4), father (2), sister (2), niece (1), daughter-in-law (1), current partner (3), friend (15) and work colleague (2). There were more than 23 different relationships described, because some participants had known multiple survivors. The majority of participants were women (18), most were white (including ‘White British’, ‘White European’ and ‘White Other’ ethnicities) and their ages ranged from mid-20s to 80.

### Procedures and data collection

People who were interested in taking part, having seen the posters or online advertisements for the study, contacted the first author (AG) by telephone or email. They were given a study information leaflet and a copy of the consent form (via email or mail) at least 48 hours prior to participating in an interview. Written consent was obtained from each participant. For safety, face-to-face interviews took place in university buildings or community premises (eg, private rooms in local council offices). Participants also had the option to be interviewed over the telephone or using Skype. Only AG and each participant were present during the interviews, and participants were only interviewed once. Sociodemographic data were collected to inform the analysis, contextualise participants and guide recruitment strategies. Participant confidentiality and anonymity were of paramount importance, thus only AG knew who had participated in the study. Transcripts of the data were cleaned to remove identifying information prior to sharing with the team for analysis, and all data were held securely in accordance with University of Bristol regulations. The limits of confidentiality, particularly reporting requirements for safeguarding issues, were explained to participants. To reduce the likelihood of distress, the voluntary nature of the research was emphasised throughout, and the researcher was attentive to participants' emotional state.

The interviews were conducted between August 2012 and April 2013 by the first author. A topic guide (which had been pilot tested) was used, with questions and prompts to elicit information pertinent to the research question. In addition, a form of member-checking was undertaken by AG throughout the interviews, by restating and summarising information to check accuracy of understanding with participants. Interviews were audio-recorded, transcribed *verbatim* and imported into NVivo10 software. The interviews ranged in length from 35 to 90 min, and saturation of themes was reached after 23 interviews. Following the interviews, an information sheet detailing local and national DV services and counselling services was shared with participants.

### Researcher reflexivity

Part of ensuring the rigour of qualitative research is for investigators to recognise that they themselves necessarily form part of the context for interactions with participants, and that they bring their traditions, values and personal qualities to each aspect of the study.^36^ For this reason, it is also important for the reader to have an understanding of *who* conducted the research: the first author (AG) is a woman, white and was in her late 30s when she carried out the interviews as part of her PhD. She had been a senior research associate for 4 years prior to her PhD studentship, and continued to work as a counsellor alongside her research (participants were informed that AG was a PhD student, but not that she had an additional counselling role). AG had had no prior contact with participants. Reflexivity also involves an active noticing by the researcher as she journeys through the research process, which for this study included keeping a reflective diary and detailed fieldnotes to capture reflections on: context, interview process, thoughts about participants, and about the relationships created during the interviews. At the interview stage, the recording of these reflections helped AG to consider what had gone well and what could have been performed differently, in order to hone interview skills and use of the topic guide. At the analysis stage, the noted descriptions of key messages from the interviews were revisited, in order to check that the developed themes reflected these.

### Data analysis

A thematic analysis of the data was carried out and was undertaken in parallel with the interviews. In thematic analysis, transcripts are read multiple times in conjunction with fieldnotes, and key concepts noted.^37^ These concepts form a list of initial codes which are applied line-by-line to the transcripts (for this study, using NVivo10 software). Initial descriptive codes are grouped into themes which were refined using *constant comparison*: a process throughout the analysis of comparing units of data with the entire data set and emerging theories, to modify constructs and relationships between them.^38^ For this study, AG analysed all of the transcripts, and EW and GF each analysed a subset. The researchers familiarised themselves with the data, identifying text that was relevant to the research question. AG generated initial descriptive codes, a vast index to encompass everything that might be of interest. AG then collated linked codes in tentative groupings at the broader level of themes. The themes were honed, through discussion, until consensus between the authors was reached, and any relationships between the coded data were noted—this was an iterative process which distilled and refined in a cyclical fashion.^39^

In the end stages, fieldnotes were revisited to check whether the honed themes reflected the key messages recorded immediately postinterview.

In the presentation of findings, illustrative quotes from participants' narratives are used. The parentheses after each quote contain the participant's pseudonym and their relationship to the survivor.

## Findings

The generated themes described a variety of different types of impact on health and well-being experienced by informal supporters of survivors. For clarity, the impacts on psychological and emotional well-being have been split into two sections. The first describes the impacts people experienced following the witnessing (either visually or by description) of incidents, such as shock, fear and panic. The second relates to impacts that were connected with the overall strain and pressure of the situation, including: anger, frustration, anxiety, distress, sadness, confusion and guilt. In the final section of the findings, the impacts on physical health are described; where the stress of the situation had begun to take a toll on people's functioning and physiology.

### Psychological and emotional impacts

A large theme that emerged from the interviews was the impact on psychological and emotional well-being, which one participant described as the *emotional burden.* People talked about the recurrence or persistence of these impacts, with several suffering ill-effects long after abusive relationships had ended. Many of the impacts were experienced concurrently or in succession; thus, there was a cumulative effect on people's well-being.

#### Impacts following the witnessing of incidents

##### Shock and horror

Several participants spoke of their shock when they first heard about the abuse, witnessed it first-hand or witnessed the aftermath. For a few participants, this shock was particularly triggered by seeing survivors' injuries following physical violence, or by unfolding revelations about the extent of the violence.

##### Fear and panic

This shock, at what the perpetrator was capable of, could lead to fear and panic, in response to a sense of threat that participants felt for their own safety: Suzie (a mother to a survivor) spoke about how frightened she was when the perpetrator was threatening to kill *her*. For Vicky, it was a growing sense that the perpetrator was a very dangerous man:

I thought, ‘If he's worked out that I'm interfering and trying to pull her away from him, trying to help her to escape, he may well do anything irrational to me to stop me from interfering.’…I had to really train myself to remember that the bogey man wasn't there, [the perpetrator] wasn't there, I'd parked my car, there was nobody around, walk with purpose, be confident, he's not gonna attack you. (Vicky, Work colleague)

For other people, fear was linked with situations they recognised as highly dangerous for the survivor. These fears were proportional and realistic about potential outcomes, including: the abduction or harming of children and the death or serious injury of the woman. Emily described how panic could ensue during periods when the survivor's future was in doubt:

I was kind of living on adrenalin, I was sort of just walking from room to room. I couldn't sit down, I couldn't concentrate. My mind was just racing, I was just in a state of panic. (Emily, Mother)

#### Impacts resulting from the overall strain of the situation

##### Anger and frustration

Most participants talked about feelings of anger and frustration. For people who felt these emotions, the predominant cause was the perpetrators' behaviour towards survivors and children:

I felt this anger welling up inside of me, and I just felt that I needed to sort of move away from him. … It's building up, and I can feel it. I just feel that, I mean I want to go round there and give him a good hiding, and I'm 70. (Eric, Father)

Several people also mentioned anger towards professionals or relatives, who they felt had responded insufficiently. Often tied with these feelings was a strong sense of injustice; that what was *right* had not prevailed:

I feel very angry that no one helped her. And now I know that it was Social Services' responsibility to help the child and to help her. It was their responsibility… I still feel angry, because I think the way they did it, the baby could have died, they were putting the baby at risk. (Zakia, Friend)

For many, there was nothing short-lived about their anger, particularly where the perpetrator continued to be abusive towards the survivor via his contact with their children. In addition to anger, people often mentioned a level of frustration they felt towards the survivor, largely when they believed she was not using her capacity to act.

##### Anxiety and worry

All of the participants described feeling anxious or worried about the situation, and for many people, these feelings, and the associated thoughts, pervaded their lives for a period of weeks, months or even years. Some people described worry in the initial stages of the relationship, before they knew about the abuse, which manifested as nagging concern:

It was when we were on holiday and I saw how he was towards my granddaughter that I was very worried, and when we came home I said to my husband that I was very concerned… (Eve, Mother)

Others described anxiety about their interactions with the survivor, or the perpetrator, wanting to guard against making the situation worse. People also mentioned ongoing concerns they had for the survivor after the abusive relationship ended, particularly the continuing potential for harm:

I still worry now that he'll hurt her, I don't ever feel 100% that something bad isn't gonna happen. (Gwen, Sister)

Fear, following exposure to abuse, could manifest as anxiety longer term, as people began to imagine all the possible outcomes of the situation. This was true for Emily, who had feared that her daughter would re-enter the abusive relationship:

I was just pacing the floor, just crying, just hysterical, I was like close to the edge. I couldn't go to work, I had to take weeks off work, ‘cos I couldn't focus, I couldn't go to work; I was just beside myself, absolutely beside myself. I really thought that there was a possibility my daughter would end up dead, if she went back, to that relationship. (Emily, Mother)

For many participants, anxious thoughts had persisted, particularly where the survivor was viewed as vulnerable, for example: by being young, by living far from their support network or by having recently exited the relationship.

##### Distress and upset

The feelings of distress and upset that people described were sometimes connected with changes in their relationship with the survivor, and sometimes with thoughts about the abuse the survivor had suffered. For Stacey, it was her friend Hannah's decision to remain in the relationship that was incredibly upsetting:

I haven't been able to contact her, because it's just too upsetting to me… ‘He's now hurting you. How's it gone from there to there?’ And then I've told her, and then that's all I can do. I can't do anymore ‘cos I'm just so upset. (Stacey, Friend)

In describing what her team of colleagues had been exposed to, Vicky spoke metaphorically of a *little container of terrible distress*, an awfulness that was not easy to shake, due to the nature and frequency of abuse their colleague suffered.

Several participants talked about the longevity of distress. Suzie, for example, spoke of continued pain evoked by memories of harrowing times while supporting her daughter. People who had been in an abusive relationship themselves, or who had been exposed to DV during childhood, spoke of their distress as memories of their own past resurfaced:

To watch it happening to somebody else I found very distressing…I was very frightened of my father at that age. (Lily, Friend)

##### Overwhelm and saturation

Some participants spoke about having reached a point where they felt overwhelmed or saturated, using words like, *breaking point*, *exhausted* and *drained* to convey the all-consuming nature of the situation. Others described peaks and troughs of intensity, and the need to take time out on occasion, to protect their own sense of well-being.

##### Tension and turmoil

Linked with feelings of shock, that some people experienced when they first heard about the situation, several participants also described the longer term challenges to their core beliefs about: humanity, justice and safety in the world. The way people described these impacts intimated the unsettling nature of having foundational assumptions called into question. Josie discovered that three women she knew had been abused by partners, which challenged her ideas about DV not happening to women who were professional or *strong*. Lily also struggled with the idea that her intelligent and dynamic friend chose to remain in an abusive relationship, and Emily was unsettled by the idea that DV could happen to people who were like her. For others, it was the fact that the survivor was prepared to remain with a violent man that led to their *bewilderment*:

I didn't know how people could live like that, how you could treat someone like that, or even how you could go back to someone after they'd treated you like that. (Anne, Friend)

Many participants also described inner dissonance; conflicting pressures within themselves, leaving them ill-at-ease. Before they had understood the situation, Sally and Eric experienced tension between their love for their daughter, and frustration at the way Amanda was behaving towards them. A few participants also spoke of the tension between the desire to intervene and the need to respect the survivor's wishes:

She had her plan and we wanted to respect that. But the stress that came with not hiring a van, going there, dealing with him … the stress of that was monumental at times. (Louise, Friend)

##### Sense of responsibility

Some participants found themselves in a position of feeling a *burden,* a *duty* or a *weight* of responsibility because of the nature of the situation. These people spoke of putting their own priorities on hold, of substantially altering life-plans and of the all-consuming nature of supporting a survivor through intense periods. Where there was complexity in the situation, the sense of responsibility was compounded; for example, where the survivor had an addiction, had children with the perpetrator, had a mental health condition or where she lacked additional social support.

##### Feeling disempowered

Another description which appeared in people's narratives was disempowerment. Participants spoke about feeling impotent to intervene during the relationship, and to protect and support sufficiently in the aftermath:

I felt really helpless that she was going back to situations where we knew she was gonna be hurt, but by then understanding domestic violence, knowing that for her safety that's what she wanted to do. And we only had to go with what she wanted … (Gwen, Sister)

Several people spoke about the persistence of this sense of powerlessness; that months or years after the end of abusive relationship, they still felt unable to stop the perpetrator impacting on the lives of their loved ones:

I just feel as if I want to protect my daughter and my grandchildren … it's very, very painful, very painful. But I don't seem to be able to do anything about it. My hands are tied and I need to get her out of this mess. (Eric, Father)

There was also a sense that some people lacked voice; that their experiences and their viewpoints were often disregarded, seen as unimportant or invalidated. Silencing came in many forms; sometimes it was professionals or employers not acting on information, and sometimes the survivor herself, either intentionally or unwittingly, prevented expression. Occasionally, participants silenced themselves by questioning the legitimacy of *their* feelings:

I do [get opportunity to voice those thoughts] a bit, but I guess to some extent I feel that I should be supportive of Judy, because she is the victim and I kind of think I should just be able to be a bit more detached, not feel that way myself, and just be there to support her. (Richard, Partner)

##### Sadness and depression

Many participants spoke of having felt low at some point; most of these people described a dip in mood that indicated despondency or a temporary sense of hopelessness, but some had been diagnosed with depression, taken antidepressants or had had suicidal thoughts. Suzie mentioned taking antidepressants at a point where she had started to feel numb:

I just I remember sitting in an armchair in my living room, literally with the duvet over me and I just couldn't move or I just lost it, I didn't really feel anything and then depression … (Suzie, Mother)

During this time, Suzie considered ending her life, because the circumstances felt so desperate. Likewise, Sally hit a similar point where she could not see a way forward:

I decided I'd kill myself (crying) … I felt just done with everything; I was just going to jump in the sea … I remember going, choosing the place. (Sally, Mother)

##### Confusion and uncertainty

All participants described periods of confusion, not only about the situation itself, and what the trajectory might be, but also about how to best support the survivor and protect themselves. At the point where people knew very little, they described feeling *in the dark* and *trying to work it out*; a piece-meal process to draw their own conclusions about the relationship, which they often discovered were inaccurate or partial:

I thought perhaps I'd upset them in some way and I wasn't sure what or how … my assumption was that they had financial troubles, and I was trying to probe to see what it was … I was worried about her. But I didn't know what I was worried about. (Barry, Father)

Stacey made the point that with health conditions, it was possible to have some sense of trajectory and outcome, unlike DV:

I think if you have a friend who's got cancer or diabetes or something like, you kinda know what's happening next…But when you're supporting someone who's in a violent relationship, you don't really know when it's gonna end, how long they're gonna need you to support them, or how much worse it's gonna get. (Stacey, Friend)

##### Guilt and self-blame

The most frequent causes of guilt described by informal supporters were not having known sooner about the DV, and not having understood what the behaviours they had witnessed meant:

I'm sad, that we couldn't help her sooner, or that we didn't prevent it from happening, it makes me sort of sad with myself really, I think, and angry at myself and, for not being supportive sooner, and doubting her. (Gwen, Sister)

Several people also described guilt they felt in relation to offering support that felt inferior. This was especially the case where their relationship with the survivor had become strained, or was lost completely. For Kate, a sense of guilt, which had persisted for many years, was her over-riding emotion:

I felt really guilty about that … I didn't feel like I could be honest with her anymore… I felt bad about it. Which was horrible of me, I still feel I've been horrible to her, because I didn't, well I don't know if I did the right thing, I still don't know if I did the right thing. (Kate, Friend)

For others, there were feelings of guilt when positive things happened in their own lives, for example, Anne described feelings akin to *survivor guilt* because she had fled an abusive relationship, started a new relationship, and become a mother, while her friend Sarah remained with her partner, and had been coerced into having an abortion.

### Physical health impacts

In addition to psychological and emotional impacts, many people talked about the *stress* of the situation; a summary term, which they used to describe some of the physical health impacts they experienced.

#### Physical symptoms and ailments

Mostly, the health repercussions participants mentioned were those which had resulted from heightened states of panic, anxiety, fear, powerlessness and anger, describing feeling sick, shaky and physically unsettled:

For me that comes with a physical feeling of almost not being able to breathe and feeling churned up inside… (Suzie, Mother)

A few people mentioned less transient physical ailments that they felt had resulted from the stress of supporting a survivor: back and neck tension, migraines, shortness of breath and tight-chestedness. Eric, in particular, felt his symptoms (similar to those of a heart attack) were connected with the anger and powerlessness he felt.

#### Sleep difficulties

Friends and relatives of survivors described broken sleep for a period of time, linked with relentless concerns for the survivor, or worries regarding their role. Relatives and partners, in particular, reported loss of sleep at critical times:

I was close to breaking point, I didn't sleep. … And I thought, this means she'll go back to him, and I remember I didn't sleep at all that week, I was just pacing the floor. (Emily, Mother)

People who mentioned sleep difficulties talked about the impact of late evening communication with the survivor, or with others involved. Some proposed an association between reduction in quality of sleep and the intense emotions experienced.

#### Appetite and weight loss

Mark and Emily mentioned loss of appetite and weight loss when discussing their health, describing it as their bodies' default response to stressful events. For Mark, it was triggered when he tried to relieve the pressure on his wife by dealing with reams of solicitor correspondence. For Emily, it happened during a time of huge anxiety, while trying to persuade her daughter from returning to the perpetrator.

## Discussion

The interviews highlighted that impacts on health and well-being of informal supporters of DV survivors were many and varied. There was a spectrum of experience in terms of severity and longevity of impact, with informal supporters describing different impacts from one another, and also changes in impact at different stages in their individual journeys. The identification of subgroups of participants with differing experiences was complex, for example, while the relationship between the informal supporter and the survivor was important, it was not whether they were relatives, friends or colleagues, but rather the quality of the relationship which mattered. The gender of the informal supporter, whether or not the survivor had children, and the level of abuse the informal supporter knew about were additional mediators of impact. Further research is needed for a greater understanding of how variance in the DV situation and in the characteristics of informal supporters influence impact.

Many of these impacts, such as anger, fear, sadness, helplessness and disruptions to sleep and to core beliefs, are sequelae of trauma; the same symptoms as those known to be experienced by people following *direct* exposure to traumatic events.^40^ ^41^

One of the suggested mechanisms through which traumatic experiences have health implications is the stress-process framework.^42^ ^43^ Within this framework, external stressors provoke physiological and psychological responses,^43^ ^44^ which impact on health and well-being, particularly if the stressors are over a long period. Given that the average length of an abusive relationship is 5 years,^45^ those involved are certainly at risk of chronic stress and its sequelae. More than 20 years ago, Figley^46^ ^47^ suggested that these effects were not limited to the person experiencing traumatic events; that emergency responders and therapists could also be affected, particularly when repeatedly exposed to incidents or disclosures over time. More recently, changes to the DSM-5 have drawn attention to those providing *informal* support as well as those providing professional support.^25^ The findings from this study add weight to the idea of risk of STS for people providing informal support in the particular scenario of DV. In addition, research suggests differential experiences of traumatic stress dependent on factors such as personal characteristics, sociodemography, social support and aggregate life events.^42^ ^43^ ^46^ ^48^ The variation in reported impacts (in terms of type, severity and longevity) by participants in this study lends support to this idea.

Moreover, there is overlap between the findings from this study, and research with people providing informal support to relatives or friends who have experienced other forms of trauma. For example, one in three spouses of Holocaust survivors were found to be suffering from psychological distress and STS symptoms,^49^ and Christiansen *et al*^24^ reported that relatives, friends and partners of men and women who had been raped showed *‘significant levels of traumatization’*, with 25% suffering from PTSD.

### Implications for policy, practice and research

The findings from this research indicate that the health and well-being of informal supporters are affected in situations of DV. In terms of policy, the social context of survivors is rarely visible, which needs to be addressed, so that the needs of informal supporters are considered. In addition, there is need for professionals who work in positions where they routinely come into contact with survivors to attend to other people within the situation; reflecting on who might be experiencing impact, and providing opportunities for disclosure, and for legitimisation of concerns. Healthcare providers, in particular, are well placed to respond to all parties affected by DV, which is why training around this issue for doctors, nurses and allied health professionals is vital.^50–52^

Research about informal supporters is crucial for understanding the context of survivors' lives.^53^ Specifically, with the intention of improving outcomes for informal supporters *and* for survivors, research is needed to develop and test interventions directly targeting those in the social networks of survivors.

### Strengths and limitations

One of the limitations of this research is that the sample lacked breadth for certain sociodemographic characteristics, ethnicity in particular. People from minority ethnic backgrounds are frequently under-represented in research^29^ and, while substantial effort was made to recruit people from a variety of ethnic backgrounds, this was not especially successful. Moreover, though a wide definition of DV was used (to include perpetrators who were other family members), the experiences captured were almost exclusively those of informal supporters of survivors of intimate partner violence. The reported findings relate specifically to this sample, so it is possible that the experiences of other people providing informal support to a survivor would differ.

A key strength of this study is the novelty of perspective because it accessed the experiences of informal supporters of survivors directly, which is vital in order to understand the wider context and implications of DV.

## Conclusion

Research has drawn attention to the extent to which women experiencing violence seek support from their friends, colleagues and family members, and the advantages this can have for their well-being and safety. The impact that this has on the health and well-being of people providing informal support has previously been unexplored. Findings from this study indicate the physical, psychological and emotional impacts on people providing informal support, suggesting that this is a group of people who may be at risk of STS. In order to prevent and reduce these impacts, informal supporters of survivors would benefit from recognition of their predicament, and provision of support, so that their own well-being, quality of life, capacity and coping are not diminished. These findings have practical and policy implications, so that the experiences and needs of the full range of people in DV scenarios are legitimised and met.
