# Correction: Qualitative study to explore the health and well-being impacts on adults providing informal support to female domestic violence survivors

**DOI:** 10.1136/bmjopen-2016-014511corr1

**Published:** 2019-05-30

Gregory A, Feder G, Taket A, *et al.* Qualitative study to explore the health and well-being impacts on adults providing informal support to female domestic violence survivors. *BMJ Open* 2017;7:e014511. doi: 10.1136/bmjopen-2016-014511

This article was previously published with an error in the acknowledgement.

At present the acknowledgement reads:

‘The authors would like to acknowledge and sincerely thank all the participants who took part in this research.’

The correct acknowledgement is:

‘The authors would like to acknowledge and sincerely thank all the participants who took part in this research. Thanks are also extended to the National Institute for Health Research (NIHR) School for Primary Care Research, and the Elizabeth Blackwell Institute for Health Research supported by the Wellcome Trust (grant number 105612/Z/14/Z), for the funding received.’

